# Hearing Outcomes of Infants Born to Mothers With Active COVID-19 Infection

**DOI:** 10.7759/cureus.25571

**Published:** 2022-06-01

**Authors:** Panagiota Kosmidou, Ioannis Karamatzanis, Sotiris Tzifas, Aggeliki Vervenioti, Despoina Gkentzi, Gabriel Dimitriou

**Affiliations:** 1 Otolaryngology - Head and Neck Surgery, Mediterranean Hospital of Cyprus, Limassol, CYP; 2 Otolaryngology, University of Patras, Medical School, Patras, GRC; 3 Internal Medicine, University of Nicosia Medical School, Nicosia, CYP; 4 Pediatric, General University Hospital of Patras, Patra, GRC; 5 Pediatrics, University of Patras, Patras, GRC; 6 Pediatrics, Infectious Diseases, Univesity Hospital Patras, Patras, GRC; 7 Pediatrics, University of Patras, Medical School, Patras, GRC

**Keywords:** coronavirus vertical transmission, newborn hearing screening, pediatric hearing loss, transient evoked otoacoustic emissions, covid 19

## Abstract

Introduction

COVID-19, caused by SARS-CoV-2, is a highly contagious respiratory tract infection. A major concern of SARS-CoV-2 infection in pregnant women is vertical maternal-fetal transmission and the ramifications on infant hearing. This retrospective study aims to investigate whether perinatal exposure to SARS-CoV-2 has an impact on the hearing of the offspring.

Materials

The study population included neonates born to unvaccinated COVID-19 positive mothers in the University Hospital of Patras, Greece from March 2020 to January 2021. Polymerase chain reaction (PCR) tests were performed on the neonates on the first, second,^,^ and seventh day of life. All neonates underwent transient evoked otoacoustic emissions (TEOAEs) within the first three months of life and were all examined at the age of nine months.

Results

Thirty-two neonates (21 male) were born within the study period and all were transferred to the Neonatal Intensive Care Unit (NICU). Their mean (SD) gestational age was 36.9 (+2.23) weeks and their birth weight was 2,943 (+537) g. Nine of them were preterm and six of them had a low birth weight. Apgar scores calculated at 1’ and 5’, were in the normal range for 31 (97%) out of 32 neonates. One infant required urgent intubation at birth with an Apgar score of 1’ 3 and 5’ 4. Four neonates required mechanical ventilatory support, two neonates required nasal CPAP and eight neonates required supplementary oxygen. All infants were negative for TORCH infections.

PCR tests were performed within the first day of life and repeated at 48 hours and on the seventh day of life. All PCR tests came back negative. Out of 32 neonates, seven failed the TEOAE test and were tested again a month later with a positive outcome. At nine months of follow-up, all 32 infants passed the TEOAE test.

Conclusion

In conclusion, in our study, there was no evidence of vertical transmission of SARS-CoV-2 from mothers infected during the third trimester or hearing impairment of the offspring.

## Introduction

The identification of the first case of coronavirus disease 2019 (COVID-19) in the Wuhan province of China in December 2019, marked an important landmark in modern history. COVID-19 is caused by severe acute respiratory syndrome coronavirus 2 (SARS-CoV-2), a highly contagious respiratory tract infection declared a pandemic on March 11, 2020, by the World Health Organization [1].

COVID-19 is an acute respiratory tract infection, subdivided into mild to moderate, severe, and critical types [2]. Symptoms of COVID-19 include fever, cough, myalgias, fatigue, and dyspnea [3]. Disease manifestations can vary significantly, from a cough or sore throat to acute respiratory distress syndrome (ARDS), respiratory failure, and death [4]. COVID-19 disease course shows acute progression, and the current lack of therapy allows for the infection to become life-threatening [5].

A major concern of SARS-CoV-2 infection in pregnant women is a vertical maternal-fetal transmission. Although intrauterine transmission is possible from a pathophysiological standpoint, actual in-utero infections appear to be rare, estimated at 2% [6]. Infection outcomes during pregnancy were divided according to the trimester in which the mother was infected: first, second and third trimester. Symptoms are similar in all trimesters, with fever and cough reported as the most common [7]. However, fever during the first trimester is potentially dangerous. The first trimester is the period of organogenesis, and hyperthermia has been linked to the death of dividing neuroblasts, disruption of cell migration, and vascular damage [8].

The first three months post-conception are the most sensitive period for ear development as many of the inner and middle ear structures begin to develop at this stage [9]. Despite this, ear development is a dynamic process that is completed by the term [10]. COVID-19 is more prevalent in the third trimester and damage in late pregnancy predisposes the child to infections or ototoxic insults [11]. Sensorineural hearing loss is one of the most common and severe complications of intrauterine exposure to certain viruses such as cytomegalovirus (CMV), rubella, and others [12].

In our study, all newborns born to mothers that had COVID-19 infection during delivery underwent audiological assessments, known as transient evoked otoacoustic emissions (TEOAEs). The study aims to investigate whether perinatal exposure to SARS-CoV-2 has an impact on the hearing of the offspring.

## Materials and methods

A retrospective study conducted in a tertiary level NICU

The Institutional Review Board (IRB) of the University of Patras Medical School, Patras, Greece, issued approval 722/19-11-2018 for this retrospective study. Consent was acquired by all mothers involved in this study, and data confidentiality and protection were maintained throughout the study.

The study population included 32 neonates born to unvaccinated COVID-19 positive mothers in the University Hospital of Patra, Greece from March 2020 to December 2020. In order to test for the presence of COVID-19 in children, three polymerase chain reaction (PCR) tests were conducted. The tests were undertaken on the first day, after 48 hours, and on the seventh day.

**Figure 1:**
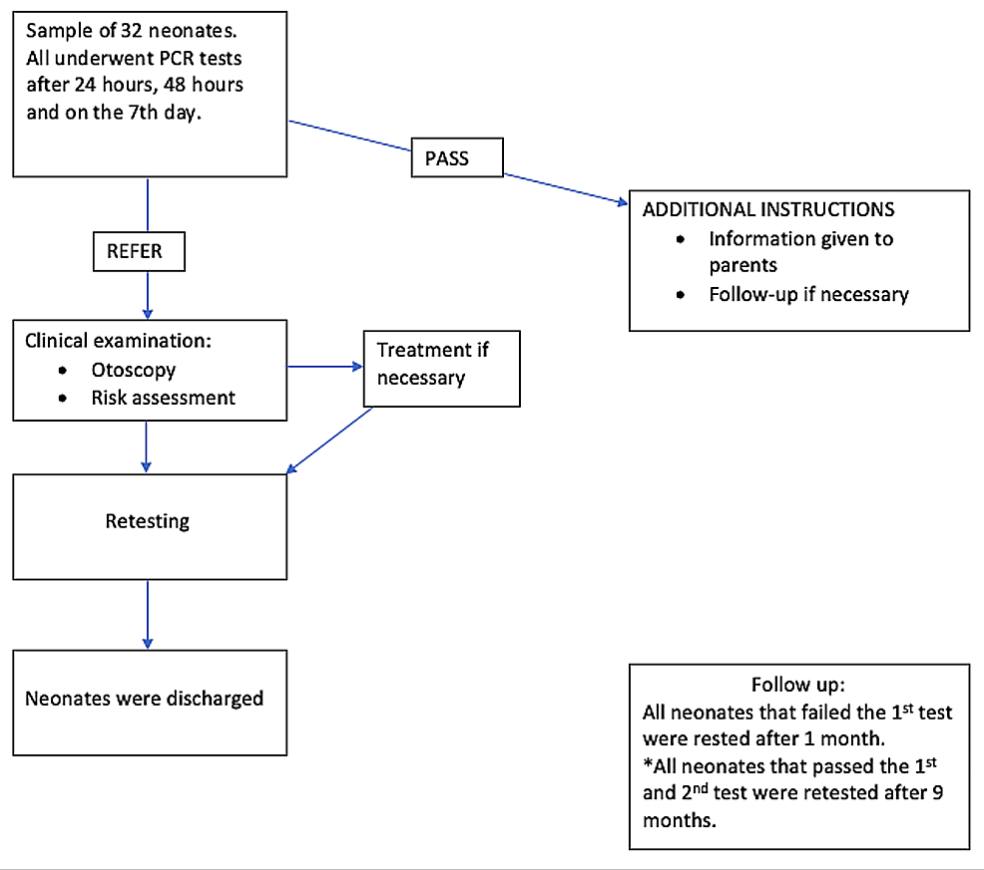
Pathway of the procedure followed in the study.

To evaluate the auditory function of the newborns, TEOAE was performed within the first three months of life and were all examined at the age of nine months (Figure 1). However, the neonates that failed the first test were retested within the first month. Failure of both the first and second TEOAE tests led to a referral and further investigations at audiology centers. The study adheres to the Joint Committee of Infant Hearing guidelines which include hearing screening completion by one month of age, diagnosis of any hearing loss by three months of age, and entry into early intervention by six months of age [13].

## Results

Thirty-two neonates (21 male) were born within the study period and all were transferred to the Neonatal Intensive Care Unit (NICU) for PCR tests to be performed as their infection status was unknown. Their mean (SD) gestational age was 36.9 (+2.23) weeks and their birth weight was 2,943 (+537) g (Table 1). Nine of them were preterm, and six had a low birth weight. Apgar scores calculated at 1’ and 5’, were in the normal range (>7 ) for 31 (97%) out of 32 neonates. One infant required urgent intubation at birth with an Apgar score of 1’ 3 and 5’ 4. Four neonates required mechanical ventilatory support, two neonates required nasal continuous positive airway pressure (CPAP) and eight neonates required supplementary oxygen. All infants were negative for toxoplasmosis, other agents, rubella, CMV, and herpes (TORCH) simplex infections.

**Table 1:** Characteristics of the neonates born to COVID-19 positive mothers at birth

Number	Gender	Date of Birth (weeks)	Birth Weight (g)	Apgar Score ( 1’ and 5‘ minutes)	Severity of Maternal Disease	Positive for COVID-19
1	M	36	2,710	7-9	Moderate	Νο
2	F	37	3,060	9-10	Mild	Νο
3	F	34	1,920	9-10	Severe	Νο
4	M	33	2,060	9-10	Moderate	Νο
5	M	37	3,500	7-9	Mild	Νο
6	M	31	1,600	7-9	Severe	Νο
7	M	38	2,700	9-10	Mild	Νο
8	F	34	2,700	7-9	Moderate	Νο
9	F	36	2,790	8-9	Severe	Νο
10	M	34	2,270	3-4	Severe	Νο
11	M	37	3,410	9-10	Mild	Νο
12	M	41	3,160	9-10	Mild	Νο
13	M	40	3,310	8-9	Mild	Νο
14	M	39	3,440	9-10	Moderate	Νο
15	F	39	3,220	8-9	Mild	Νο
16	M	36	2,440	8-9	Moderate	Νο
17	M	38	2,940	9-10	Mild	Νο
18	M	38	3,940	9-10	Severe	Νο
19	F	37	2,630	6-8	Mild	Νο
20	M	33	2,190	8-9	Severe	Νο
21	M	37	3020	8……9	Mild	Νο
22	F	37	3550	9……10	Mild	Νο
23	F	37	2570	9…….10	Mild	Νο
24	F	40	2940	6…..8	Mild	Νο
25	M	38	3310	9……..10	Mild	Νο
26	M	39	3750	9…….10	Mild	Νο
27	F	39	3280	8…….9	Mild	Νο
28	M	37	3150	5………8	Moderate	Νο
29	F	38	3260	9…….10	Mild	Νο
30	M	38	3090	9…….10	Mild	Νο
31	M	37	2980	9…….10	Mild	Νο
32	M	37	3270	9…….10	Moderate	Νο

**Figure 2:**
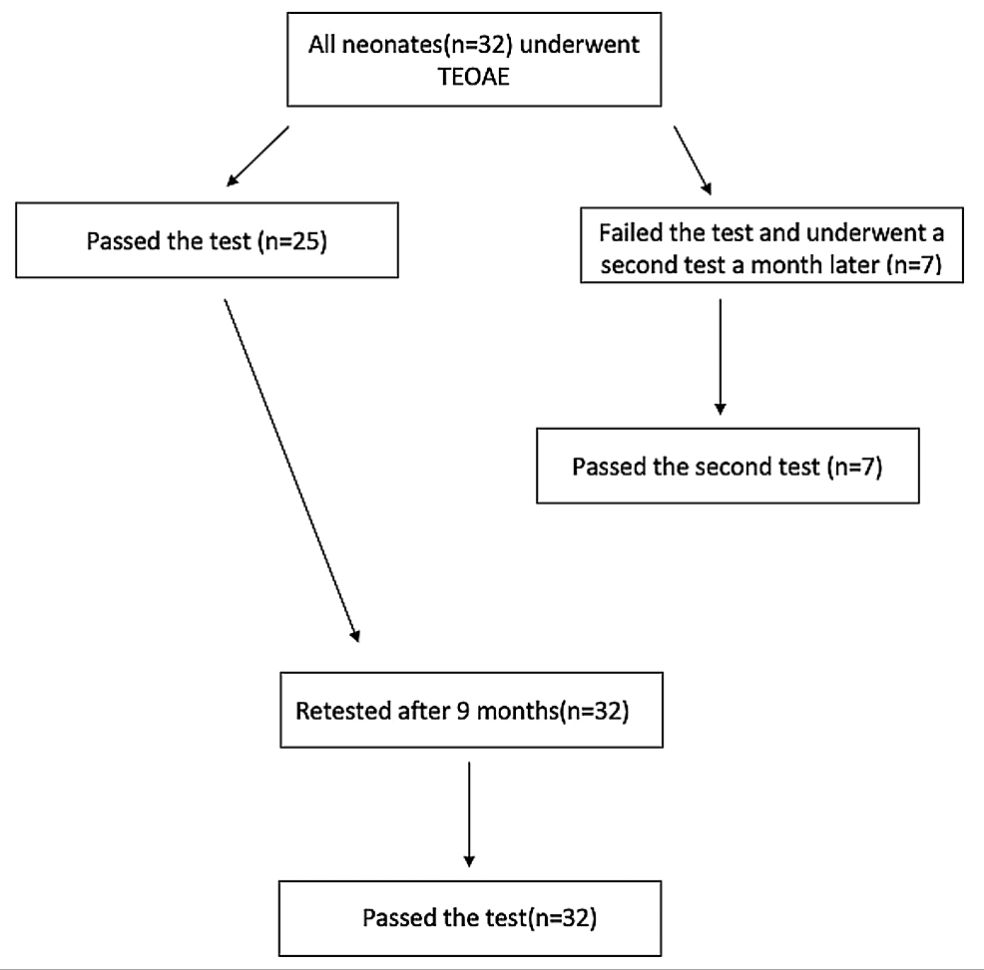
Illustration of the TEOAE screening process of neonates. TEOAE - transient evoked otoacoustic emission

PCR tests were performed within the first day of life and repeated at 48 hours and on the seventh day. All PCR tests came back negative. Out of 32 neonates, seven failed the TEOAE test and repeated the test a month later with a positive outcome. At the nine-month follow-up, all 32 infants passed the TEOAE test (Figure 2).

Regarding the mothers, 19 suffered mildly from COVID-19, seven suffered moderately and five had severe complications. It is important to note that all mothers suffering from moderate disease received oxygen and all mothers suffering from severe disease were intubated. Of note, all of them were unvaccinated as the vaccine has not been authorized for pregnant women during the study period.

## Discussion

COVID-19 is an acute respiratory tract infection with one of the most common outcomes being pneumonia [2]. Pneumonia is the most common cause of fatal non-obstetric infections in pregnant women [14]. Pneumonia affects both the mother and fetus and has been linked to increased complications, compared to pregnancies in which infection is absent [15]. Studies found that pregnant women with pneumonia had a higher likelihood to have preterm deliveries, lower birth weight, and small gestational age infants, than women without pneumonia [15]. It is important to note that physiologic adaptations that occur during gestation predispose pregnant women toward a more severe course of pneumonia [14]. Immunological changes, most prevalent in the second and third trimesters, such as reduced number of T-cells, decreased cell-mediated cytotoxicity, and overall diminished lymphocyte proliferative response, all lead to an increased risk of complications from pneumonia [14]. 

Reverse transcriptase-PCR (RT-PCR) is considered to be the gold standard for the diagnosis of COVID-19 and found to have almost perfect specificity [16]. However, RT-PCR is not faultless. The low sensitivity of the test and a considerable number of false negatives resulted in individuals unintentionally spreading the disease [17]. Reports of suspected cases that tested negative with the PCR test were later found to be undiagnosed [18]. 

COVID-19 has been compared to a TORCH infection as they demonstrate similar incidence. Both COVID-19 and TORCH infections show higher infection rates in the third trimester [19]. Hearing loss caused by viruses can be mild or severe to profound, unilateral, or bilateral. Viruses can directly harm inner ear structures, including inner ear hair cells and the organ of Corti, as well as act indirectly through host-immune response [20]. The hematogenous spread of SARS-CoV-2 to the central nervous system is via ACE2 receptors [21]. ACE2 receptors are expressed on glial tissues, neurons, and brain vasculature which make them a target for SARS-CoV-2 [21]. Extrapolating from TORCH infections, COVID-19 has the potential for neurological damage in the inner ear. For example, CMV is the leading cause of non-genetic congenital sensorineural hearing loss (CSNHL) and CSNHL is the most common outcome of congenital rubella infections [22]. As hearing loss is a common sequela of in-utero infections, many scientists worry about the long-term effects of COVID-19.

Moreover, the trimester that the mother contracted SARS-CoV-2 is significant for fetal outcomes. The severity of disease outcomes for the fetus has an inverse correlation with gestational age, as first-trimester infections produce more harmful effects [10]. Embryologically, the first trimester is the period of organogenesis, the most sensitive period for significant congenital malformations [23]. Additionally, intrauterine infection with COVID-19 has been linked to increased rates of fetal growth restriction, preterm birth, and perinatal mortality [24]. A possible explanation by Alan and Alan is that fetal growth restriction, preterm birth, and perinatal mortality result in delayed neuron maturation which affects hearing screening results [25]. Therefore COVID-19 is a plausible, albeit transient, a risk factor for hearing loss. Furthermore, the direct viral-induced cytokine storm and general pro-inflammatory state can negatively impact fetal brain development and may lead to a broad spectrum of neurologic sequela [26].

The TEOAE test is used as a screening tool for the detection of hearing loss in infants. Neonates born to COVID-19 positive mothers underwent TEOAE tests. The otoacoustic emissions produced in the cochlea are recordings of the mechanical capacity and mobility of the outer hair cells [27]. The TEOAEs screening is reported >90% sensitive (80%-96.5%), and specific (90.60%-92.85%) [28]. The Joint Committee of Infant Hearing (JCIH) states known risk factors for early childhood hearing loss. These include but are not limited to the use of ototoxic drugs for more than five days, stay in the neonatal intensive care unit (NICU) for more than five days, family history of hearing loss, in utero infections and low Apgar scores [13]. Despite of all the risk factors, all neonates in the study were negative for COVID-19 and all passed the TEOAE test. 

Furthermore, in the study by Ghiselli et al., only one child (1.9%) had bilateral refer results [29]. The above results are consistent with the JCHI indications that describe a reference standard of less than 4% for newborns that do not pass the first step of screening. In a recent study, Veeranna et al. found that COVID-19 during pregnancy may not be a risk factor for hearing loss, as they reported no difference in the Auditory Brainstem Response (ABR) thresholds [30]. In contrast, Alan and Alan found that children born to COVID-19 positive mothers had a significantly higher risk of having abnormal hearing screening results [26].

There was no documented vertical transmission in our sample mission as none of the newborns were positive even though pregnant mothers were unvaccinated and found positive for COVID-19. However, according to the JCIH guidelines, the monitoring frequency of children exposed to in utero infections is specified “as per concerns of on-going surveillance” [13]. The study aims to continue surveillance of these children for research purposes. 

Our study had limitations within which our findings have to be interpreted. First, the study was conducted in a single center and thus our findings may not be applicable to other hospitals. Another limitation is the lack of a control group to compare our findings, which was not possible due to the pandemic. Lastly, the small size of the sample is also another factor that restricts the generalization of the findings.

## Conclusions

In conclusion, in our study, there was no evidence of vertical transmission of SARS-CoV-2 from mothers infected during the third trimester or hearing impairment of the offspring. Strict adherence to NICU protocols and close follow-up is the gold standard for early detection of neonatal hearing loss and the prevention of its complications. Finally, the question remains whether COVID-19 should be included as a risk factor for congenital hearing loss.
